# Configural Analysis in Component Space

**DOI:** 10.17505/jpor.2022.24217

**Published:** 2022-06-09

**Authors:** Alexander von Eye, Wolfgang Wiedermann

**Affiliations:** 1 Michigan State University; 2 University of Missouri, Columbia

**Keywords:** Configural Frequency Analysis, Principal Component Analysis, Sectors of component space, configural analysis of multiple variables

## Abstract

Unless very large samples are available, the number of variables and variable categories that can be simultaneously used in categorical data analysis is small when models are estimated. In this article, an approach is proposed that can help remedy this problem. Specifically, it is proposed to perform, in a first step, principal component analysis or factor analysis. These methods help reduce the dimensionality of the data space without loss of important information. In a second step, sectors are created in the component or factor space. These sectors can, in a third step, be subjected to Configural Frequency analysis (CFA). CFA identifies those sectors that contradict a priori-specified hypotheses. It is also proposed to take into account the ordinal nature of the sectors. In addition, distributional assumptions can be considered. This is illustrated in data examples. Possible extensions of the proposed approach are discussed.

Configural Frequency Analysis (CFA; Lienert, 1968; von Eye & Gutiérrez Peña, 2004; von Eye & Wiedermann, 2021) allows researchers to answer the question whether patterns of variable categories, known as *configurations*, were observed more often than expected, less often than expected, or about as often as expected, all under a particular probability model. Configurations are said to *constitute a CFA type*, when the observed number exceeds the expected number. They are said to *constitute a CFA antitype* when the observed number is smaller than the expected number.^1^

CFA is applied to cross-classifications of categorical variables. This characteristic implies that either the number of variables and their categories are relatively small or the sample that is studied is very large. In empirical studies, researchers usually have comparatively small samples available (see von Eye, & Wiedermann, 2021). Large samples are rare, very large samples are very rare. To the best of our knowledge, there exists only one case in which a sample with millions of cases was subjected to (a form of) CFA (DuMouchel, 1999).

In addition, even when the number of categorical variables and their categories are relatively small, continuous variables cannot be subjected to CFA. The cross-classification of continuous variables would be far too large to be analyzed with CFA. Therefore, data analysts often categorize variables before data analysis. This procedure (also called *binning*) reduces the size of tables often considerably, but it comes with a price, that is, loss of information.

In this article, we propose a new approach to the problem that larger numbers of variables and continuous variables cannot be analyzed with CFA. Specifically, we propose a three-step procedure. The first step involves subjecting continuous variables to principal component analysis or factor analysis. The second step involves creating sectors in the component or factor space that results from the first step. In the third step, CFA is used to analyze the sectors that result from the second step.

The new approach is a generalization of a method that was proposed to determine the sectors in a factor space that deviate from multinormality (von Eye, & Gardiner, 2004). Here, this method is extended to accommodate the goals of CFA. The new approach is also a generalization of a method that was recently proposed by von Eye and Wiedermann (under review). This approach allows researchers to perform CFA under consideration of the scale level of binned variables (i.e., the ordinal nature of binned variables) and possible multinormality. Here, in contrast, observed continuous variables are neither binned nor crossed. Instead, the component or factor space is split in sectors that can be subjected to CFA. The contribution of the present article is to elaborate a method that allows researchers to perform CFA on multiple continuous and categorical variables of various characteristics.

This article is structured as follows. In the next section, we illustrate the procedure of creating sectors in a data space. In the following sections, we briefly review CFA (for more detail, see von Eye & Gutiérrez Peña, 2004; von Eye & Wiedermann, 2021) and principal components analysis (PCA). In the section following this, we show how to segment the component space and to perform CFA on the resulting sectors.

## Creating sectors in a data space

In the remainder of this article, we focus on the component space. This is the space that uses the components that result from PCA as axes. Alternatively, factor spaces could be used or, when the number of continuous variables is small, the space that is spanned by observed variables themselves. Let *d* be the number of components, with *d* ≥ 1. The creation of sectors in this *d*-dimensional space follows the steps that are performed when the Chi-square test is used to test whether a single variable is normally distributed. These steps are discussed in many introductory statistics textbooks (e.g., Glass & Hopkins, 1984). Taking an algorithmic perspective, the sectors can be created in the following two steps (cf. von Eye & Bogat, 2005; von Eye & Gardiner, 2004):

1. Split each of the *d* components into two or more segments. Thus, component *j* will have *cj* segments, with *j* = 1, …, *d*;
2. Cross the segmented components to obtain a cross-classification with *Π_j_C_j_* sectors.

To illustrate, we use the responses from a self-declared male alcoholic who participated in a prospective longitudinal study on the development of alcoholism (Perrine et al., 1995). This individual provided responses on a series of questions on 733 consecutive days. These questions included, among others, the number of beers, hard liquor, glasses of wine, and cigarettes consumed the day before the interview, the amount of stress experienced, mood, subjective health, and an overall rating of the day. These eight variables are hard to analyze with standard CFA. Just consider that each of these responses is split into three segments. The cross-classification of these eight categorized variables would contain 3^8^ = 6561 cells. For this large table, even the many responses given by this respondent would be insufficient. On average, each cell would contain no more than 0.11 responses.

**Figure 1:**
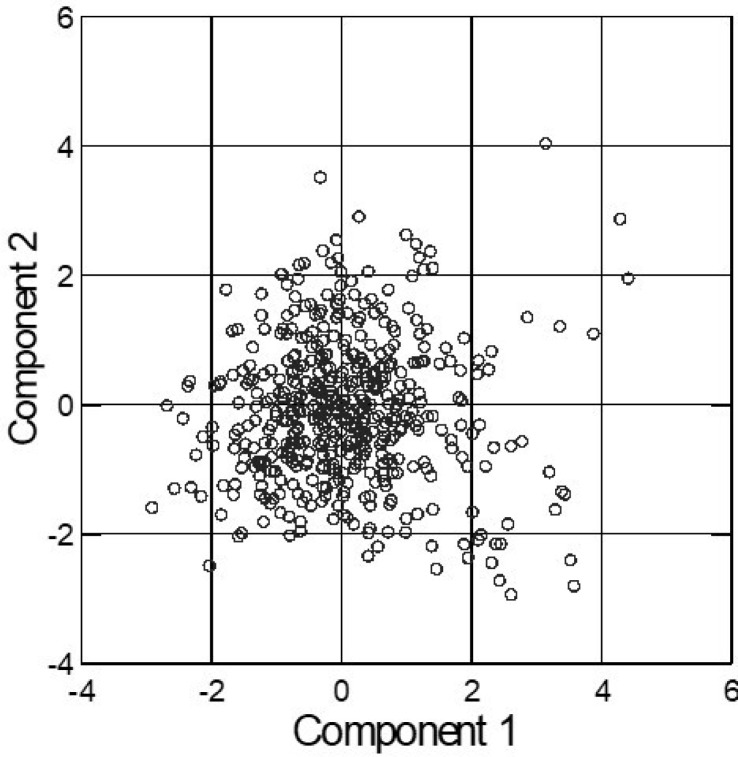
Scatterplot of two principal components with five sectors each.

We now perform a PCA on the eight variables. This PCA results in two components. To create the sectors, we split the two components in five segments each. Crossed, the scatterplot of the components looks as given in Figure 1.

Figure 1 shows that the majority of the responses can be located near the origin of the component space. This is under the assumption of a bivariate normal distribution of the component scores, as expected. The method proposed by von Eye and Gardiner (2004; see also von Eye & Bogat, 2005) can be used to test whether there are deviations from this distribution. Evidently, in this example, there is a number of responses that are extreme on one or both axes. This issue is discussed in the context of CFA, later in this article.

## Configural Frequency Analysis

In this section, we give a brief overview of CFA (for more detail, see von Eye & Gutiérrez Peña, 2004; von Eye & Wiedermann, 2021). Consider a cross-classification of *d* completely crossed categorical variables. The cell frequencies in this cross-classification are estimated based on a data generation process (DGP; von Eye, Wiedermann, & von Weber, 2021). From this process, probability models are derived, the so-called *base models*, in which *none* of the hypotheses of interest is part of the effects that are considered. Therefore, when the process is rejected, at least some of these hypotheses are bound to exist, statistically.

In contrast to variable-oriented methods of analysis such as log-linear modeling that focus on variable relations, CFA focuses on individual cells, that is, configurations. For each configuration, the observed cell frequency is compared with the expected cell frequency. As was mentioned above, when more cases are observed than expected, the configuration is said to constitute a *CFA type*. When fewer cases are observed than expected, the configuration is said to constitute a *CFA antitype*.

To test the null hypothesis of no type or antitype, either tests from residual analysis of log-linear models can be employed, or tests that were developed specifically for CFA (for an overview of the former, see von Eye & Wiedermann, 2021; for an example of the latter, see von Eye & Mair, 2008). These tests are either exact or approximative, they either can be used under any sampling scheme or require product-multinomial sampling, and, depending on sample size, they can differ considerably in power. Usually, CFA tests are applied to many cells of a cross-classification. Therefore, in the domain of frequentist statistical inference, the protection of the significance threshold *α* is de rigeur.

CFA is conducted in the following four steps. The first step is the specification of a *base model*. This is a model that is derived from the a priori-specified DGP. The second step involves the selection of a significance test. The third step is the selection of a procedure for α protection. The fourth step involves the estimation of expected cell frequencies, the comparison of observed and expected cell frequencies and the interpretation of CFA types and antitypes.

In the next section, we briefly review principal component analysis.

## Principal Component Analysis

Principal component analysis (PCA; Pearson, 1901) is widely known as a method that can be used for dimension reduction, without loss of important information. The method is used to reduce *t* variables to *d* components, with *d < t*. The *d* - 1^st^ component is orthogonal to all components before. Most important for the purposes of the present article is that the results of PCA are considered useful in particular when, in subsequent steps, other multivariate statistical methods are applied (see Raykov & Markoulides, 2008). For that, there is no need to substantively interpret the components.

PCA is performed in the following steps (see Jaadi, 2021).

(1) *Standardization of raw data*: each of the *t* variables is standardized. This step is necessary to prevent variables that differ in variance from making unequal contributions to the final solution (alternatively, the correlation matrix can be analyzed instead of the covariance matrix);

(2) *Calculation of the t × t covariance matrix, **X**, of the observed variables*. PCA uses this matrix for further analysis.

(3) *Calculation of the eigenvectors and eigenvalues of the covariance matrix with the aim of identification of components*. The right eigenvector of ***X, R***, satisfies ***XR = λ_R_R***, where ***λ_R_*** is the eigenvalue of the right eigenvector. The left eigenvector of ***X, L***, satisfies ***LX = λ_L_L***, where ***λ_L_*** is the eigenvalue of the left eigenvector. Both ***R*** and ***L*** are diagonal matrices. When ***X*** is symmetrical, which is the case for a covariance or a correlation matrix, then ***C*** = ***LR*** is diagonal as well, ***L*** and ***R*** are the transpose of each other, and, for the scalars ***λ_L_*** and ***λ_R_***, it holds that ***λ_L_*** = ***λ_R_*** ≡ ***λ***. The ***λ***s are the *eigenvalues*. They indicate the amount of variance retained by the corresponding components (eigenvectors). The first eigenvector represents the largest portion of variance of the original data. The subsequent eigenvectors represent decreasingly less variance. The *feature vector* is a matrix that contains the *d* eigenvectors the researcher wishes to keep and use.

(4). *Express the original data in terms of the feature vector.* This can be done by multiplying the transposed original data matrix by the transposed feature vector. This operation re-expresses the original data in terms of the component space that is *d*-dimensional instead of *t*-dimensional, as is the original data set.

Alternative methods of calculating component scores exist. The selection from these methods depends, for example, on whether or not the researchers wish to rotate components, desire component scores that are as close to a normal distribution as possible, desire components that are orthogonal, or wish that variances of the scores are 1 or not. In the remainder of this article, we use the method described above. This method is known as the *regression* method.

In the following section, we jointly use PCA and CFA in the sense suggested by Raykov and Marcoulides (2008). Specifically, we propose, first, performing PCA, second, creating sectors of the resulting component space, and, third, performing CFA on the components of the sector space.

## CFA of the sectors from a PCA

To create sectors in the component space, we adapt the method proposed by von Eye and Gardiner (2004) who discussed sectors in a *normal linear factor space* (Bartholomew et al., 2002). The component space can be created to have the following characteristics (for characteristics of the normal linear factor space, see Bartholomew, et al., 2002):

(1) the components are uncorrelated with each other (data analysts relax this characteristic when they perform oblique rotation of components);

(2) the components have a mean of zero and a variance of one;

(3) the residuals are uncorrelated with each other and with the components;

(4) the residuals have a mean of zero but can have different variances (when ***X*** is not standardized);

(5) the component scores follow a multivariate normal distribution (depending on how they are calculated and on how well the components represent ***X***).

Violations of the last characteristic can have various reasons. One reason is the presence of outliers (see Figure 1). Another reason is the shape of the distribution of the component scores (see bullet 5; Yuan, & Bentler, 2001). A third reason is that the raw data in ***X*** stem from non-normal populations. von Eye & Gardiner (2004) propose methods that allow the researcher to identify the sectors that contain more or fewer cases than expected under the assumption of a normal distribution (see also von Eye & Wiedermann, under review). Here, we jointly apply the method of identifying out-lying sectors and CFA.

To create sectors in the component space, consider *d* components that create a *d*-variate distribution of component scores. These scores are calculated as described in the last section, on PCA. Now, let the range of scores of component *m* be divided in *c_m_* segments. Then, the space of the *d* crossed components has $\Pi_{m=1}^{d}c_{m}$ sectors. The number of segments for each component is determined based on the sample size and the desired resolution of the subsequently applied CFA.

### CFA of component scores

Most CFA base models are log-linear models of the form $\hat{m}=X\lambda$, where $\hat{m}$ is the vector of estimated cell frequencies (also called *model frequencies*), *X* is the design matrix, and ***λ*** is the parameter vector. *X* contains the effects that represent the *CFA base model*. This model represents all effects that are not of interest to the researcher. If this model fails, the effects of interest are bound to statistically exist. In addition, for failing models, some of the cells (configurations) can constitute *CFA types* or *CFA antitypes*.

CFA base models that are not log-linear do exist. However, they are of interest only in specific cases, and they are rarely used. Examples of such cases can be found in von Eye and Wiedermann (2021). These models are outside the scope of this article. Here, we consider base models of the form

$$\log\;\hat{m}=[1|X_{s}]\left[\begin{array}{c} \lambda_{1}\\ \lambda_{s} \end{array}\right],$$

where **1** represents the model constant, that is, a vector of 1s in the design matrix, used to estimate the model intercept, and *X*_s_ contains the part of the model that represents the sectors of the segmented components. Unless the components are subjected to an oblique rotation, *X*_c_, contains just the main effects of the components.

We now give an example of a CFA of a sectored component space. In this example, we continue the analysis of the data that were used for Figure 1. The 25 sectors are subjected to a first order CFA. We perform the four steps of CFA.

*Step 1: Specification of base model.* The base model for this CFA is the one given above, that is,

$$\log\;\hat{m}=[1|X_{s}]\left[\begin{array}{c} \lambda_{1}\\ \lambda_{s} \end{array}\right],$$

or, expressed in terms of the effects in this model, $\log\;\hat{m}=\lambda +\lambda^{C1}+\lambda^{C2}$, where *C1* and *C2* are the two segmented components of PCA. When this model fails to well describe the data in Figure 1 and in Table 1 (below), there may be sectors in which more or fewer cases are found than expected under the assumption of independence of the two components. In the present example, the components are split into five segments of equal length. The first segment begins with the lowest score and the last segment ends with the largest score.

**Table 1:** First order CFA of the two components of an alcoholic’s responses

Configuration				
*C*_1_ *C*_2_	*m*	$\hat{m}$	*p*	
11	5.00	2.7534	.14478289	
12	23.00	15.2316	.03612895	
13	14.00	20.3869	.08765653	
14	1.00	4.3937	.06608557	
15	.00	.2343	.79106919	
21	9.00	23.2439	.00059623	Antitype
22	108.00	128.5831	.02372470	
23	204.00	172.1035	.00357491	
24	41.00	37.0913	.27716455	
25	1.00	1.9782	.41157701	
31	19.00	17.9932	.43710466	
32	113.00	99.5368	.08286101	
33	117.00	133.2262	.06427071	
34	31.00	28.7125	.35716602	
35	1.00	1.5313	.54720928	
41	11.00	2.3052	.00002870	Type
42	13.00	12.7520	.51013082	
43	11.00	17.0681	.07981952	
44	1.00	3.6785	.11756099	
45	.00	.1962	.82183839	
51	3.00	.7044	.03459785	
52	3.00	3.8965	.45347800	
53	2.00	5.2153	.10680884	
54	1.00	1.1240	.69022506	
55	2.00	.0599	.00172435	Type

*Step 2: Selection of significance test*. In this example, as can be seen in Figure 1, some of the sectors contain just a few cases, or none. Therefore, we select the binomial test, that is, an exact test that can be applied under any sampling scheme.

*Step 3: Selection of procedure for the protection of α.* Here, we select the procedure that was proposed by Holland and Di Ponzio Copenhaver (1987). In comparison to the well known Bonferroni procedure, the one by Holland and Di Ponzio Copenhaver (1987) results in less extreme protected thresholds.

*Step 4: Performing CFA and interpreting types and antitypes*. Table 1 displays the results of this CFA.

The overall goodness-of-fit of the base model is poor (LR-*X^2^* = 76.850 *df* = 16; *p* < 0.001). We, therefore, expect that types and antitypes emerge. Table 1 suggests that two types and one antitype exist. The types are constituted by Sectors 4 1 and 5 5. The antitype is constituted by Sector 2 1.

It is an interesting characteristic of sector CFA of PCA components that the interpretation of types and antitypes cannot resort to semantic characteristics of the components. PCA usually is employed to reduce the dimensionality of variables. In contrast to factor analysis, there is no semantic interpretation of factors. Reduction of space is the main aim, not the identification of interpretable factors. All we note is that the two components explain 28.655 + 14.562 percent of the total variance, that is, in all, 43.217%.

One may ask whether adding a third component would be useful in the sense that a larger portion of variance is accounted for. In the present example, however, the scree test suggests using the two-component solution. The elbow of the scree plot is strongly prominent between two and three components.

From CFA, we realize that the two components are not independent. There are two sectors in which significantly more responses are found than expected under the hypothesis of component independence, and there is one sector in which significantly fewer responses are found than expected under this hypothesis. We conclude that

(1) the PCA solution explains only a relatively small portion of the overall variance, and

(2) the postulate of independence of PCA components is violated in three of the 25 sectors created by partitioning the two components in five segments each.

In the present article, we propose applying log-linear base models to the cross-classification of the segments of components of a PCA of multiple variables. Any base model can be used, and the sector-component space can also be crossed with such categorical variables as type of vehicle, diagnose of disease, or gender. When sectors are crossed with categorical variables, the base model becomes

$$\log\;\hat{m}=[1|X_{s}|X_{c}]\left[\begin{array}{c} \lambda_{1}\\ \lambda_{s}\\ \lambda_{c} \end{array}\right]$$

where *X_c_* indicates the vectors that represent the effects that are considered for the additional categorical variables, and *λ_c_* are the corresponding parameters. *X_c_* contains at least the main effects of the categorical variables. Higher order effects require theoretical justification. When there are no effects that link the components and the additional categorical variables, this base model is one of Prediction CFA.

In the present article, we do not follow this line of base model development. Instead, we now ask, how, under the assumption of a *d*-variate normal distribution, the probability that an object is located in one of these sectors can be calculated. To answer this question, we adapt the methods proposed by von Eye and Gardiner (2004) and von Eye and Wiedermann (under review).

### CFA of component sectors under multinormality

We first consider the univariate case, that is, the case in which there is just one PCA component. Let *z_m_* be the standardized component score of the lower limit of segment *m*. The first segment then begins at *z_1_* = -∞. Correspondingly, the last segment ends at $z_{c_{m}}=+\infty$ Then, the probability for a component score to be located in the segment between *z_m_* and *z_m+1_* is

$$p(z_{m+1})-p(z_{m})=\int_{-\infty }^{z_{m+1}}\Psi (z)dz-\int_{-\infty }^{z_{m}}\Psi (z)dz$$

This is the area under the normal curve, Ψ, in the segment that is bounded by *z_m_* and *z_m+1_*.

In the multivariate case, the probability for an element to be located in the sector that is bounded by $z_{i}^{1}$ and $z_{i+1}^{1}$ on variable 1, $z_{j}^{2}$ and $z_{j+1}^{2}$ on variable 2, …, and $z_{k}^{d}$ and $z_{k+1}^{d}$ on varialbe *d*, where the subscripts index the segments and the superscripts index the variables, is

$$\begin{array}{l} \;p\left(z_{i}^{1}-z_{i+1}^{1},z_{j}^{2}-z_{j+1}^{2},\ldots ,z_{k}^{d}-z_{k+1}^{d}\right)\\ =\int_{z_{i}^{1}}^{z_{i+1}^{1}}\int_{z_{j}^{2}}^{z_{j+1}^{2}}\ldots \int_{z_{k}^{d}}^{z_{k+1}^{d}}\Psi (z^{1},z^{2},\ldots ,z^{d})dz^{1}dz^{2}\ldots dz^{d} \end{array}$$

(cf. Genz, 1992; for estimating the probability for convex sectors, see Somerville, 1998), where Ψ indicates, as before, the area of the normal distribution. In what follows, we abbreviate this probability with *p_i, j,…,k_*. The corresponding sectors are denoted by *s_i,j,…,k_*.

The expected frequency of objects in Sector *s_i,j,…,k_* is ${\hat{e}}_{i,j,\ldots ,k}=Np_{i,j,\ldots ,k}$, where *N* is the sample size. von Eye and Gardiner (2004) proceeded, from this point on, as follows. They proposed, to identify sectors in which multinormality is violated, comparing, in each Sector *s_i,j,…,k_*, the observed frequency of objects, *m_i,j,…,k_*, with the expected frequency, ${\hat{e}}_{i,j,\ldots ,k}$, under the null hypothesis that $E[m_{i,j,\ldots ,k}]={\hat{e}}_{i,j,\ldots ,k}$. When this comparison suggests that a sector contains significantly more or fewer objects than expected based on the joint density function of the *d* variables under study, this sector evinces a violation of multivariate normality. Therefore, the assumption of multivariate normality must be rejected at least for this sector.

Here, we move in a different direction. Keeping the null hypothesis unchanged, we propose considering three approaches to estimating the expected sector frequencies, ${\hat{e}}_{i,j,\ldots ,k}$. The first of these approaches incorporates the method proposed by von Eye and Gardiner (2004). This method allows the researcher to answer the questions whether (i) the component scores are multinormally distributed, and (ii), if not, which sectors stand out by containing too many or too few cases. These questions are of importance in particular when component scores were estimated using methods that are supposed to result in multinormal component scores, but also in standard methods, e.g., the regression method described above. This approach can be viewed as analogous to Wiedermann and von Eye’s (2016) approach in which they ask whether local independence exists in latent classes.

The second approach considers the ordinal (interval) level nature of the segments created for each component. Naturally, the segments created on a component range from low to high on the scale that is used to segment the component are ordinal even when the segments are not evenly spaced. Here, we use Goodman’s (1979, 1894, 1991) linear-by-linear association model that was also used by von Eye and Wiedermann (under review). Not using the information that is carried by the ordinal nature of the component segments can result in unnecessarily complex models.

The last approach combines the three approaches previously used in this article, that is, the approach of segmenting components, the approach of taking distributional characteristics of component scores into account, and the approach of taking the ordinal nature of segments into account.

### Data example of CFA of component scores under multinormality

In this section, we illustrate the analysis of sectored components under the hypothesis of multinormality. To this effect, we employ the method proposed by von Eye and Wiedermann (under review; cf. von Eye & Gardiner, 2004). We continue the analysis of the above data example. To estimate the sector-specific bivariate normal probabilities, we use Somerville’s (1998) algorithm. The resulting probabilities are (from Sector 1 1 through Sector 5 5) 0.004324, 0.027888, 0.033776, 0.007755, 0.000317, 0.025478, 0.164319, 0.199015, 0.045696, 0.001871, 0.024469, 0.157811, 0.191138, 0.043888, 0.001797, 0.003820, 0.024640, 0.029844, 0.006853, 0.000281, 0.000089, 0.000571, 0.000691, 0.000159, and 0.000007. We now perform the four steps of CFA.

*Step 1: Specification of base model*. The base model that takes these probabilities into account is

$$\log\;\hat{m}=[1|X_{s}|X_{c}]\left[\begin{array}{c} \lambda_{1}\\ \lambda_{s}\\ \lambda_{n} \end{array}\right],$$

where *X_s_* represents the main effects of the two components, and subscript n indicates the bivariate normal probabilities. In the present example, the covariate is hypothesized to be independent of the main effects of the two categorized components. This model corresponds to a model of first order CFA with covariate *X_n_* in which the covariate is hypothesized to be independent of the main effects of the two categorical variables. If this model fails to describe the data well, the two components are related to each other, relations among these main effects and the bivariate normal probabilities exist, statistically, or both. Types and antitypes will, then, indicate where these effects are most prominently visible.

*Step 2: Selection of significance test.* To be able to compare this analysis with the one in Table 1, we select, again, the binomial test.

*Step 3: Selection of procedure for the protection of α.* Also as before, we select the procedure of Holland and Di Ponzio Copenhaver.

*Step 4: Performing CFA and interpreting types and antitypes.* Table 2 contains the results of this CFA.

**Table 2:** First order CFA of sectored data of an alcoholic’s responses with the covariate bivariate normality of the component scores.

Configuration				
*C*_1_	*C*_2_	*m*	$\hat{m}$	*p*
1	1	5	5.2482	0.42765830
1	2	23	14.1174	0.01720805
1	3	14	15.5377	0.41009288
1	4	1	7.5983	0.00418122
1	5	0	0.4984	0.39257152
2	1	9	19.7752	0.00522375
2	2	108	130.0454	0.01696646
2	3	204	178.9555	0.01846990
2	4	41	32.6109	0.08226669
2	5	1	1.6130	0.47947860
3	1	19	15.9633	0.25275163
3	2	113	100.5954	0.10200376
3	3	117	136.9675	0.03073052
3	4	31	26.1622	0.19156068
3	5	1	1.3116	0.37736608
4	1	11	4.4693	0.00618686
4	2	13	11.7692	0.39744612
4	3	11	12.8847	0.36323991
4	4	1	6.4508	0.01152419
4	5	0	0.4259	0.34692334
5	1	3	1.5439	0.20213818
5	2	3	3.4725	0.45766034
5	3	2	3.6546	0.29247568
5	4	1	2.1778	0.35958313
5	5	2	0.1511	0.01032187

The overall goodness-of-fit of the base model is poor (LR- *X^2^* = 62.625, *df* = 15; *p* < 0.001). We, therefore, expect that types and antitypes emerge. Table 2 suggests, however, that neither types nor antitypes exist. Evidently, we encounter, again, the situation in which the overall goodness-of-fit of the base model suggests rejecting the model, but none of the discrepancies between individual observed and expected cell frequencies is large enough to result in types and antitypes. We, therefore, conclude that considering distributional characteristics can change the results of CFA considerably.

Clearly, this would apply to the results of log-linear modeling as well (see von Eye, & Wiedermann, under review). The model under the hypothesis of a normal distribution is significantly better than the model without this hypothesis (ΔLR- *X^2^* = 14.225, Δ*df* = 1; *p* < 0.001). Still because of the poor overall fit of the base model, we cannot interpret the parameters (the parameter for the normal distribution vector would have made a significant contribution).

## Discussion

In this article, the development of CFA is advanced by

1. deriving an approach that allows one to perform CFA of multiple continuous variables; this approach involves subjecting these variables to PCA, creating sectors in the space of PCA components, and analyzing the sector space using the methods of CFA; additional categorical variables can be taken into account;
2. incorporating recent data generation processes including those that result in variables that are ordinal in nature or multinormal.

In the following paragraphs, we discuss characteristics of the proposed approach. The first concerns the application of PCA. PCA cannot be automatically used when there are many more variables from one behavior domain than another. Over-represented variables can have the effect that components reflect this weight. Therefore, data analysts may wish to make sure that variables and variable groups carry the intended weight.

Application of PCA filters data through a linear lens. This procedure implies that data patterns that are based on pair-wise variable relations can disappear, that is, may not be detected by CFA. Types and antitypes, therefore, may reflect higher than first order relations. In the parts of this CFA, types and antitypes can, therefore, be compared with types and antitypes from second order CFA (see von Eye & Wiedermann, 2021).

Accordingly, principal components are the weighted sums of the original variables. Therefore, component scores tend to be normally distributed to a degree that exceeds that of the original variables. However, as was noted above, higher order relations are not affected by this characteristic. Therefore, CFA of component scores can still detect sectors that contain fewer or more cases than expected under the assumptions of relations that were specified in the base model that is employed in a particular analysis (cf. von Eye & Bogat, 2005; von Eye & Gardiner, 2004).

In the examples given in this article, we applied PCA to all variables subjected to CFA. This, however, is, by no means, a necessary procedure. Researchers might consider subjecting multiple variables from specific domains to separate PCAs. For example, when intelligence is measured using multiple variables, PCA may be used just for the intelligence variables. Other variables can be subjected to separate PCAs or even used without any transformation. When this is done, types and antitypes indicate local relations among variable groups.

In either case, principal components are rarely interpreted, substantively. PCA serves to reduce the variable space. When researchers wish to interpret the dimensions of a reduced space, they may wish to perform factor analysis or, when variables are categorical, latent class analysis (Wiedermann & von Eye, 2016) rather than PCA. Either of these methods is also applicable in the present context.

Principal components are just orthogonal, new variables that represent linear combinations or mixtures of the un-transformed variables. It should be noted, however, that some researchers tend to interpret principal components just as they would interpret factors in factor analysis. In this case, the magnitude of the correlation of variables with the individual component guides interpretation. The magnitude that justifies interpretation is, as in factor analysis carried by criteria that are often subjective.

Similarly, the rules that guide the decision concerning the number of principal components are often subjective. The scree plot is used by some researchers, but there are more rules. The discussion of this topic is beyond the scope of this article. Data analysts may inspect textbooks on multivariate statistics (e.g., Raykov & Marcoulides, 2008).

We now discuss possible generalizations and extensions of the approach proposed here. The first of these concerns the method that is used to reduce the dimensionality of the variable space.

When factor analysis is used, it is important to take into account obliquely rotated factors. In this case, the segmented dimensions are related, and types and antitypes can reflect these relations. They indicate the sectors of the factor space in which the relations are most prominently visible. When moderator CFA is applied, types and antitypes can differ over the groups that are defined by moderator variables.

When latent class analysis is applied, the validity of a solution can vary over the latent classes. When, in addition, moderator variables are used, differences between latent classes can vary over the categories of the moderator variables.

Another issue of interest concerns the underlying distribution of the component or factor space, or the latent classes. In this article, we used the normal distribution as an example. Other sampling distributions could have been used a well. It is important to realize that, depending on the method used to calculate component or factor scores, not just any distribution is meaningfully applied. For example, when component or factor scores are calculated such that they approach multinormality, the uniform distribution or asymmetric distributions can lead to biased results. Therefore, in the present context, symmetric distributions such as the normal or the binomial (for, e.g., *p* = 0.5) are often the most appropriate ones. This can be different in other contexts.

A third option to extend the approach proposed here concerns the method of data analysis after reduction of the variable space. In this article, we focused on CFA. For the same cross-classifications, however, log-linear models can be estimated (cf. von Eye & Wiedermann, under review). Moving from CFA to log-linear modeling corresponds to a move from person-oriented research to variable-oriented research. CFA focuses on individual profiles. In contrast, log-linear modeling focuses on variable relations. Still, in either method, the ordinal nature of variables can be taken into account, models can be estimated for sectored data spaces, underlying distributions can be made part of a model, covariates can be considered, and a large number of hypotheses can be tested. Still, the foci of analysis are fundamentally different.

In contrast to both, LCA does not analyze cross-classifications directly. Instead, it reduces the variable space focusing, in a person-oriented way, on the creation of profiles that are optimally separated from other profiles. These profiles describe data carriers, e.g., respondents in a survey study. Complementing Wiedermann and von Eye’s (2016) approach in which CFA is used to evaluate LCA solutions with respect to the postulate of conditional independence, the present approach allows researchers to employ class membership in the function of a moderator variable. It can, then, be studied whether PCA solutions vary across classes. Types and antitypes indicate where and how strong class-specific violations are, and, when distributional assumptions are made, whether violations of such assumptions are also class-specific.

Finally, it can be discussed whether the variable space can be reduced by other methods than factor analysis and PCA. Cluster analysis is a prime option. Using cluster analysis variables can be grouped, and the relations among variables that were not part of the cluster analysis can be inspected by cluster. The clusters function, in this approach, as moderators of variable relations.

In sum, the approach proposed here is a first step toward CFA or log-linear modeling of multiple continuous variables that, thus far, prevented data analysts from using either CFA or log-linear modeling.

## Footnotes

1 Here and in the remainder of this article, ‘more than expected’ and ‘fewer than expected’ are used to indicate a statistically significant difference.

## Action editor

Lars-Gunnar Lundh served as action editor for this article.
